# A Rosaceae Family-Level Approach To Identify Loci Influencing Soluble Solids Content in Blackberry for DNA-Informed Breeding

**DOI:** 10.1534/g3.120.401449

**Published:** 2020-08-06

**Authors:** Jason D. Zurn, Mandie Driskill, Sook Jung, Dorrie Main, Melinda H. Yin, Melissa C. Clark, Lailiang Cheng, Hamid Ashrafi, Rishi Aryal, John R. Clark, Margaret Worthington, Chad E. Finn, Cameron Peace, Amy Iezzoni, Nahla Bassil

**Affiliations:** * USDA-ARS National Clonal Germplasm Repository, Corvallis, OR; † Department of Horticulture, Washington State University, Pullman, WA; ‡ Department of Horticulture, University of Arkansas, Fayetteville, AR; § School of Integrative Plant Science, Horticulture Section, Cornell, Ithaca, NY; ** Department of Horticultural Science, North Carolina State University, Raleigh, NC; †† USDA-ARS Horticultural Crops Research Laboratory, Corvallis, OR; ‡‡ Department of Horticulture, Michigan State University, East Lansing, MI

**Keywords:** Fruit sweetness, *Rubus* spp., RosBREED, Marker-assisted breeding, Marker-assisted selection

## Abstract

A Rosaceae family-level candidate gene approach was used to identify genes associated with sugar content in blackberry (*Rubus* subgenus *Rubus*). Three regions conserved among apple (*Malus × domestica*), peach (*Prunus persica*), and alpine strawberry (*Fragaria vesca*) were identified that contained previously detected sweetness-related quantitative trait loci (QTL) in at least two of the crops. Sugar related genes from these conserved regions and 789 sugar-associated apple genes were used to identify 279 *Rubus* candidate transcripts. A Hyb-Seq approach was used in conjunction with PacBio sequencing to generate haplotype level sequence information of sugar-related genes for 40 cultivars with high and low soluble solids content from the University of Arkansas and USDA blackberry breeding programs. Polymorphisms were identified relative to the ‘Hillquist’ blackberry (*R. argutus*) and ORUS 4115-3 black raspberry (*R. occidentalis*) genomes and tested for their association with soluble solids content (SSC). A total of 173 alleles were identified that were significantly (α = 0.05) associated with SSC. KASP genotyping was conducted for 92 of these alleles on a validation set of blackberries from each breeding program and 48 markers were identified that were significantly associated with SSC. One QTL, qSSC-Ruh-ch1.1, identified in both breeding programs accounted for an increase of 1.5 °Brix and the polymorphisms were detected in the intron space of a sucrose synthase gene. This discovery represents the first environmentally stable sweetness QTL identified in blackberry. The approach demonstrated in this study can be used to develop breeding tools for other crops that have not yet benefited directly from the genomics revolution.

Blackberries (*Rubus* subgenus *Rubus*) are the fourth most economically important U.S. berry crop, accounting for over $650 million in sales during 2019 (CA Strawberry Commission 2019). Cultivated blackberries are primarily hybrids of two or more *Rubus* species and belong to the family Rosaceae (Clark and Finn 2011). This family is diverse and contains many important crops such as apple (*Malus* ×*domestica*), peach (*Prunus persica*), pear (*Pyrus communis*), and strawberry (*Fragaria* ×*ananassa*). Similar to other members of the Rosaceae, blackberry is highly prized worldwide for its sweet fruit (Clark and Finn 2011; Zurn *et al.* 2018).

One of the major challenges in bringing cultivated blackberry to market is a lack of cultivars that are high in sugar content and retain firmness (Clark and Finn 2011). Marker-assisted selection (MAS) is particularly effective for evaluation of genetic potential for traits with low heritability but for which most of that genetic influence is determined by one or a few loci (Ru *et al.* 2016), such as apple fruit fructose content (Guan *et al.* 2015). Currently, blackberry lacks genomic resources such as dense genetic maps, large mapping populations, and high-throughput marker genotyping assays that are needed to fuel quantitative trait locus (QTL) discovery and DNA test development underlying MAS (Garcia-Seco *et al.* 2015; Foster *et al.* 2019). High ploidy in cultivated blackberry (2*n* = 2*x*-12*x* = 14-84) further complicates the development of genomic resources and genetic analyses (Foster *et al.* 2019). Recently, QTL related to sugar content and soluble solids content (SSC) in fruit were identified in strawberry, apple, and peach (Etienne *et al.* 2002; Zorrilla-Fontanesi *et al.* 2011; Lerceteau-Köhler *et al.* 2012; Verma *et al.* 2017). SSC is often used as a proxy for sugar content and sweetness in berry crops during breeding as the majority of soluble solids in fruit are sugars (Zorrilla-Fontanesi *et al.* 2011).

Within the Rosaceae family, a high degree of synteny is observed among species due to shared evolutionary ancestry (Vilanova *et al.* 2008; Sargent *et al.* 2009; Illa *et al.* 2011; Bushakra *et al.* 2012; Jung *et al.* 2012; Edger *et al.* 2019; Hardigan *et al.* 2020). When comparing the *Fragaria* and *Prunus* genomes, Vilanova *et al.* (2008) noted a clear pattern of synteny between the two genera. Yamamoto *et al.* (2004) showed that linkage maps of Japanese pear (*Pyrus pyrifolia*) and European pear (*P. communis*) had conserved marker order for intergeneric markers on the apple consensus map. Moreover, markers have been identified that amplify syntenic regions in *Malus*, *Fragaria*, and *Prunus* (Sargent *et al.* 2009). These early findings using linkage mapping approaches are supported by the recent sequencing and assembly of many rosaceous crop genomes (VanBuren *et al.* 2016, 2018; Hibrand Saint-Oyant *et al.* 2018; Jibran *et al.* 2018; Edger *et al.* 2019; Raymond *et al.* 2018; Linsmith *et al.* 2019; Hardigan *et al.* 2020).

Genes and pathways for sweetness and fruit ripening have been shown to be conserved within Rosaceae and other plant families (Le Dantec *et al.* 2010; Wei *et al.* 2014). Sugar transport genes play a vital role in the long-distance transport of sugar and in the allocation of sugar into source and sink cells in developing fruit (Le Dantec *et al.* 2010; Wei *et al.* 2014). Wei *et al.* (2014) identified sugar transport genes in *Malus* and found them to be conserved in *Arabidopsis* and *Vitis*. A study of peach genes identified 59 candidate genes (CGs) associated with fruit quality, including sweetness (Le Dantec *et al.* 2010). Primers were designed for 55 of these CGs and were tested in strawberry and two-thirds of them produced amplicons, demonstrating that many of the genes involved in sugar production, degradation, conversion, and transport are conserved among Rosaceae species.

The development of DNA-based genetic markers for assisting plant breeding that began in the 1980s (Xu and Crouch 2008) has changed substantially with the invention of next-generation sequencing (NGS). NGS has allowed a multitude of tools and approaches to be developed for identifying polymorphisms in DNA sequence for use in MAS. One such method commonly used to detect polymorphisms is amplicon sequencing or targeted amplicon sequencing (Fritsch *et al.* 2016; Shirasawa *et al.* 2016; Onda *et al.* 2018). Amplicon sequencing produces PCR products that flank or span a polymorphism of interest and can be used to identify polymorphisms reliably and rapidly for known regions of interest with few limitations (Fritsch *et al.* 2016). With amplicon sequencing, the user only gains insight into a single region between the forward and reverse primers (Ranjan *et al.* 2016). This limited window could exclude other polymorphisms, that could be contained on adjacent exons, introns, and neighboring genes. Moreover, amplicon sequencing can be ineffective for regions or genes with high levels of sequence divergence. Another approach used for detecting polymorphisms is RNA sequencing (RNA-Seq; Wang *et al.* 2013; Garcia-Seco *et al.* 2015; Salazar *et al.* 2015). RNA-Seq is good for capturing whole mRNA transcripts, but it can be cost-prohibitive as low-level transcripts require very deep sequencing for reliable capture (Ozsolak and Milos 2011). An alternative to the aforementioned sequencing methods is Hyb-Seq (Weitemier *et al.* 2014). Hyb-Seq can target and capture long genomic sequences that contain sequence variant information in the targeted and flanking regions. This target capture approach can and has been used to cost-effectively capture low-copy nuclear genes (Kamneva *et al.* 2017). Hyb-Seq targets and captures sequences using biotinylated RNA baits. The baits can be designed from closely related species to capture syntenic genes and regions (Weitemier *et al.* 2014; Carter *et al.* 2019). Because baits can be designed from related species and polymorphisms and corresponding flanking information can be captured, Hyb-Seq is a promising approach for blackberry given the genomic complexity associated with its interspecific hybrid nature and the lack of available genomic resources.

Many of the genes and pathways mediating sugar content are likely conserved across Rosaceae and might be useful to identify associated regions in blackberry. As such, polymorphisms associated with SSC in blackberry were identified using a homologous gene-based approach and markers were developed and validated for use in DNA-informed breeding.

## Materials and Methods

### Germplasm and Phenotyping

Blackberry crosses were made in 2011, 2012, and 2013 at the University of Arkansas System Division of Agriculture (UA) and the USDA-ARS Horticultural Crops Research Unit (HCRU) breeding programs (Supplementary Table S1; Zurn *et al.* 2018). Populations and parents developed by UA were planted at the UA Fruit Research Station (Clarksville, AR) and those developed by the USDA-ARS HCRU program were planted at Oregon State University’s Lewis-Brown Farm (Corvallis, OR). Parentage for all individuals was previously verified using a microsatellite fingerprinting set (Zurn *et al.* 2018).

Parents and offspring were evaluated for two years (2015 and 2016) for SSC. In the morning before temperatures exceeded 27°, 15 berries were harvested from each plant at the shiny-black stage. Berries were frozen following harvest until ripe berries were obtained from all plants. After all berries were collected, the 15 berries from each genotype were divided into three replicates and juiced. Frozen juice from the USDA-ARS HCRU program was sent via overnight shipping to UA where it was thawed overnight before measurement. The berry juice from each sample was measured using an Abbe Mark II refractometer (Bausch and Lomb Inc., Rochester, NY, U.S.A.). Historical SSC data for the parental germplasm and important cultivars released from each breeding program were also collected from annual breeding records. Mean SSC was calculated for each individual and 20 individuals from each of the two breeding programs with high and low SSC were chosen (Table 1), to maximize the likelihood of discovering polymorphisms associated with SSC. High SSC was defined as a mean SSC greater than 11.5 °Brix, and low SSC was a mean SSC less than or equal to 11.5 °Brix.

**Table 1:** Summary of sequenced blackberries from the University of Arkansas System Division of Agriculture (UA) and the USDA-ARS Horticultural Crops Research Unit (HCRU) breeding programs. Mean historical soluble solids content (SSC), circular consensus sequences (CCS) generated during sequencing are presented, and groupings determined via K-means clustering using markers identified from the Hillquist V1 (HV1) and *R. occidentalis* V3 (RoV3) genome assemblies

Program	Name	Mean SSC (°Brix)	No. CCS Reads	Mean CCS Read Length	Median CCS Read Length	HV1 Group	RoV3 Group
HCRU	ORUS 4647M	7.6	10,745	2,678.00	2,767	1	3
HCRU	ORUS 4540N	8.0	17,995	2,723.20	2,720	1	3
HCRU	ORUS 4647L	8.2	62	1,628.70	1,493.5	2	2
HCRU	ORUS 4647R	8.6	32,015	2,880.20	2,936	1	3
HCRU	ORUS 4647U	8.9	6,914	1,802.90	1,561	1	3
HCRU	Kotata	9.3	4,310	1,109.10	1,029	1	4
HCRU	Bassettberry	9.7	5,185	1,924.70	1,646	1	3
HCRU	Ollalie	9.7	1,640	1,118.00	1,067	2	4
HCRU	Silvan	9.7	9,542	3,116.70	3,603	1	3
HCRU	Black Diamond	10.5	4,509	2,492.60	2,369	1	3
HCRU	ORUS 1932-1	11.5	9,484	2,963.70	3,258	1	3
HCRU	Marion	12.2	5,707	1,173.30	1,095	1	4
HCRU	Nightfall	12.2	9,522	2,676.80	2,439	3	3
HCRU	Columbia Star	12.8	3,988	2,703.30	2,689.5	1	3
HCRU	Waldo	13.7	6,397	2,478.10	2,143	1	3
HCRU	ORUS 4540A	15.1	4,761	2,038.20	1,702	3	1
HCRU	ORUS 4540I	15.6	16,413	3,108.20	3,386	1	3
HCRU	ORUS 4674C	16.3	2,840	1,085.90	973.5	1	4
HCRU	ORUS 4674J	17.1	2,289	1,119.00	1,017	1	4
HCRU	ORUS 4660T	18.4	7,681	2,320.20	2,208	3	3
UA	Choctaw	7.4	130	1,662.60	1,464	2	2
UA	Comanche	7.5	22,542	3,465.50	3,758	3	1
UA	A-2562T	8.3	28,239	2,112.40	1,888	1	1
UA	APF-329	8.4	9,420	2,947.00	3,009.5	3	1
UA	A-2418T	8.6	22,640	2,878.10	2,942	1	1
UA	APF-326TN	8.6	122	1,218.30	1,152.5	2	2
UA	Cheyenne	8.6	4,332	1,061.60	977.5	2	4
UA	APF-236T	8.7	37,369	3,062.30	3,125	3	1
UA	APF-306T	8.7	6,833	2,741.90	2,679	3	1
UA	Kiowa	9.0	13,368	2,425.60	2,200	3	1
UA	A-2421	11.6	3,260	1,140.70	1,050	1	4
UA	A-2548T	11.7	2,984	1,148.80	1,054	1	4
UA	Osage	11.7	32,607	3,432.50	3,681	3	1
UA	A-2444T	11.9	27,512	2,359.00	2,081	3	1
UA	A-2552T	12.0	14,495	2,918.80	2,960	1	1
UA	Ponca	12.2	2,904	877.5	789	2	4
UA	A-2542T	12.2	23,180	2,967.50	3,005	1	1
UA	A-2496	12.3	7,112	1,815.00	1,555	1	1
UA	A-2487T	12.4	5,154	2,831.80	2,876	3	1
UA	APF-318	14.3	3,965	1,789.20	1,602	1	1

### Hyb-Seq Bait Design and Sequencing

**Figure 1:**
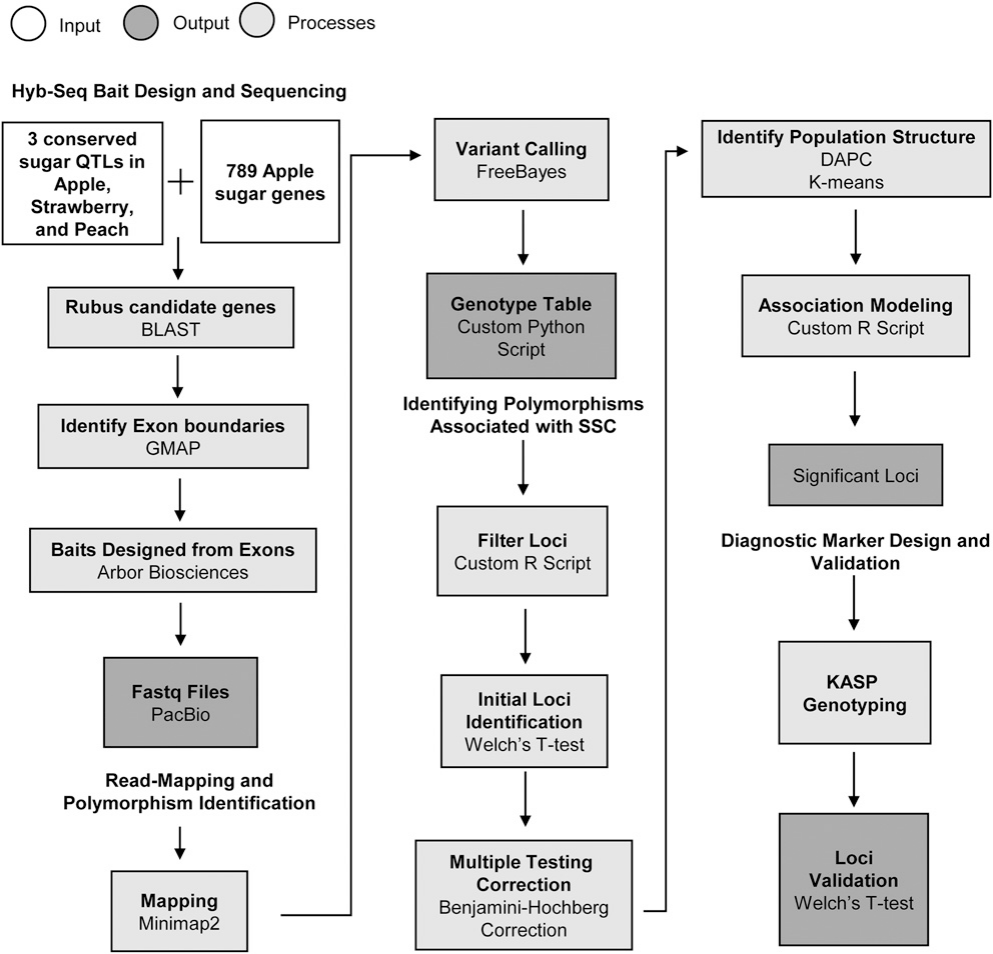
Schematic of experimental workflow.

A set of 789 unique genes from the *Malus domestica* v3.0.a1 assembly that were associated with sugar content (Li *et al.* 2012, 2016) were BLAST-searched (Altschul *et al.* 1990) against the *Rubus* RefTrans v2 transcripts from the Genome Database for Rosaceae (GDR; Jung *et al.* 2019) and filtered with an e-value cutoff of 0.01 (Figure 1). Data mining was also performed using the GDR’s tools and collated information (Jung *et al.* 2019) to identify QTL associated with sweetness-related traits for *Fragaria*, *Malus*, and *Prunus* (Quilot *et al.* 2004; Lerceteau-Köhler *et al.* 2012; Guan *et al.* 2015; Jung *et al.* 2019). The physical regions of the QTL were identified using the genomic location of SNP markers that are associated with QTL. Syntenic regions that are conserved across the *Prunus persica* v1 (Ppv1; The International Peach Genome Initiative 2013), *Fragaria vesca* v1 (Fvv1; Shulaev *et al.* 2011), and *Malus* ×*domestica* v1 primary (Mdv1; Velasco *et al.* 2010) genome assemblies, identified by the Mercator program (Dewey *et al.* 2007) and made available on GDR, were further mined to identify regions that contain sugar-related QTL from at least two of the species (Table 2). Genes were extracted from the *Fragaria vesca* v2.0.a1 genome assembly for three syntenic regions that had sugar-related QTL reported in two crops (Table 2) and BLAST2GO was used to re-annotate the extracted genes to identify those related to sugar content (Conesa and Götz 2008). *Fragaria* candidate gene sequences were BLAST-searched against the *Rubus* RefTrans v2 transcripts to identify orthologous genes in *Rubus*. Identified *Rubus* genes were mapped to the *R. occidentalis* v1.1 genome (Jibran *et al.* 2018) with GMAP version 2018-05-30 to identify intron and exon position boundaries (Wu *et al.* 2016). Exon sequences less than 50 nucleotides in length were removed and the remaining exon sequences were sent to Arbor Biosciences (Ann Arbor, MI, U.S.A.) for bait design. Arbor Biosciences designed baits to fit a 2X tiling density for the submitted exon sequences.

**Table 2:** Syntenic regions identified that contain sugar-related QTL in at least two of the investigated crops (apple, strawberry, and peach). The physical positions described are for the *Prunus persica* v1 (Ppv1; The International Peach Genome Initiative 2013), *Fragaria vesca* v1 (Fvv1; Shulaev *et al.* 2011), and *Malus ×domestica* v1 (Mdv1; Velasco *et al.* 2010) genome assemblies. References and associated markers are presented for each syntenic QTL identified

Region	Physical Positions	QTL	Crop	Associated/Delimiting Marker(s)	QTL References
1	Ppv1 scaffold_1:10507957-10972280	qFRUC.SP-G1	Peach	PC102	Quilot *et al.* (2004)
	Fvv1 LG4:18318384-19134134	SSC	Apple	ss475880868-ss475882452	Guan *et al.* (2015)
	Mdv1 Chr13:17977974-18806402				
				
2	Ppv1 scaffold_6:24867717-25235545	SSC	Strawberry	AX-89805813-AX-89906546	V. Whitaker personal communication
	Fvv1 LG6:7484789-7911306	Sucrose	Apple	ss475880518-ss475880556	Guan *et al.* (2015)
	Mdv1 Chr12:30215039-30750693				
					
3	Ppv1 scaffold_2:25475692-25774711	qSUCR.CCF-LGVIIa-f	Strawberry	BFACT044	Lerceteau-Köhler *et al.* (2012)
	Fvv1 LG7:20618518-21128712	Sorbitol	Apple	ss475876853-ss475876937	Guan *et al.* (2015)
	Mdv1 Chr1:34158601-34656509	Fructose	Apple	ss475876857-ss475876937	
		Fructose	Apple	ss475883868-ss475876937	
		Glucose-2012(20wk)	Apple	ss475876868-ss475876937	
		Glucose-2012 (10wk)	Apple	ss475876871-ss475876937	
		Sorbitol	Apple	ss475882286-ss475876937	

Young actively growing leaf tissue or the youngest possible leaf material was collected from the 40 chosen breeding selections and cultivars with low and high SSC. For individuals grown by the UA program, tissue was shipped overnight on ice to the USDA-ARS National Clonal Germplasm Repository (NCGR) in Corvallis, OR. Approximately 30-50 mg of tissue from each individual was sampled into a 96-well plate and flash-frozen in liquid nitrogen. Samples were stored at -80° until DNA extraction was conducted. Prior to extraction, samples were ground using a mixer mill (MM 301; Retsch International, Hann, Germany). DNA was extracted using the E-Z 96 Plant DNA Kit (Omega BioTek Inc., Norcross, GA, U.S.A.) following the modifications proposed by Gilmore *et al.* (2011). DNA was quantified with a Quant-iT PicoGreenTM dsDNA Assay Kit (Thermo Fisher Scientific, Waltham, MA, U.S.A.) and a Tecan Infinite M Plex multimode plate reader (Tecan Group Ltd, Zürich, Switzerland). For each sequenced sample, 1 µg of total DNA was sent to Arbor Biosciences for Hyb-Seq. Captured genomic DNA from the 40 samples were sequenced at Arbor Bioscientific using a PacBio instrument. The raw reads were processed into high-quality circular consensus reads (CCS) that were polished with the Arrow algorithm, available through PacBio tools.

### Read-Mapping and Polymorphism Identification

The CCS reads for the 40 samples were individually mapped to both the ORUS 4115-3 black raspberry *R. occidentalis* v3.0 (*R. occidentalis* v3.0, Van Buren *et al.* 2018) and the ‘Hillquist’ blackberry v1 genomes (‘Hillquist’ V1, Worthington *et al.* 2020) with Minimap2 2.15-r915-dirty, using the settings for PacBio genomic reads (Li 2018). Files generated by Minimap2 were converted to bam files, sorted, and indexed with SAMtools 1.9 (Li *et al.* 2009). The bam files for each assembly were used with Freebayes v1.2.0-4-gd15209e to identify structural variants (Garrison and Marth 2012). Freebayes was set to the recommended settings for PacBio reads with the correct ploidy reflecting each sample. A custom Python script was created to take the output VCF files from Freebayes and to create a genotype table for each reference. The tables contained loci named by the chromosome or contig, the position for each polymorphism, and the genotypic information for all 40 samples. Read depth was calculated for all positions and samples that had missing data using SAMtools 1.9 depth command. If no reads for a region were present, it was recorded as missing.

### Identifying Polymorphisms Associated With SSC

Loci identified for each assembly were filtered to have less than 20% missing data and to have between two and four alleles present across all samples. Significant loci were identified using a similar process as Wei *et al.* (2006). In Wei *et al.* (2006), markers associated with disease resistance were identified for sugarcane, which is also a complex autopolyploid like blackberry. A custom R script was used to determine the presence and absence of each locus-allele in each of the 40 samples. Each locus was initially examined individually using Welch’s T-test. A Benjamini-Hochberg correction was applied to correct for error resulting from multiple testing and to identify significant loci (α = 0.05). Each of the significant loci were fitted to two general linear models to correct for false associations due to population structure associated with each breeding program:

(1) SSC=Group+Allele+Group×Allele+Residual

(2) SSC=Group+Allele within Group+Residual

Groups describing population structure were established using a discriminant analysis of principal components (DAPC) approach based on k-means clustering using the ‘find.clusters’ function in the R package ‘adegenet’ (Jombart 2008; Jombart and Ahmed 2011). Alleles with less than 20% missing data were used for DAPC. A significant (α = 0.05) group × allele interaction would indicate the effects of the locus differed among population groups. Significant (α = 0.05) within-group variance would suggest the allele-trait association was independent of population structure. Alleles that did not have significant group × allele interactions and had significant within-group variance (*i.e.*, allele within group) were chosen for diagnostic marker design and validation.

### Diagnostic Marker Design and Validation

Two sets of 96 individuals, one from each breeding program, representing high and low SSC within each family were chosen for allele validation (Supplementary Table S1). Leaf tissue was obtained and processed as described for the PacBio sequencing and lyophilized. Lyophilized tissue and DNA consensus sequences consisting of the potential diagnostic alleles and their flanking sequences were submitted to LGC Ltd (Teddington, United Kingdom) for KASP marker design, DNA extraction, and assay execution. Diagnostic alleles were composed of the significant target allele and a second allele that could be the reference and/or an alternative allele. Due to ploidy variation, high diversity, and the complexity of genetic sequences represented by the 40 sequenced samples in a given region, some consensus sequences were designed with a preference toward the target diagnostic allele. Genotypic data were received from LGC and curated using the LGC KlusterCaller software. Alleles were validated for diagnostic ability in each environment (location-year) using Welch’s T-test and a Benjamini-Hochberg correction (α = 0.05).

### Characterization of Chromosome 1 QTL

Positions of alleles of the chromosome 1 QTL were determined using the JBrowse tool on the GDR (Jung *et al.* 2019). If an allele was determined to be in the exon or intron of a gene, the gene sequence was extracted and conserved protein domains were identified to predict gene function using the conserved domain database (Lu *et al.* 2020). Haplotype sequence information was also extracted from the Integrated Genome Viewer (IGV, Robinson *et al.* 2017) for the significant alleles. Haplotype sequences were also used in conjunction with the conserved domain database to compare and validate the results. The gene and haplotype sequences were subjected to a BLAST search to determine if similar gene functions were found in other species beyond Rosaceae

### Identification of regions in Rubus occidentalis v3.0 that are syntenic to the sugar-related QTL containing regions in peach, apple and strawberry

Synteny among the *R. occidentalis* v3.0 and the newest apple (*M*. × *domestica* GDDH13 v1.1; Daccord *et al.* 2017), peach (*P. persica* v2.0; Verde *et al.* 2013), and strawberry (*F. vesca* v4.0; Edger *et al.* 2017) genome assemblies was identified for the three conserved sugar-related QTL regions (Table 2). The synteny analysis in GDR was conducted using MCScanX (Wang *et al.* 2012) with default settings. The genomic sequences from old genome assemblies of apple, peach and strawberry (Table 2) were first BLAST-searched to the corresponding new genomes, then the syntenic regions in *R. occidentalis* v3.0 was identified using the synteny browser in GDR (Jung *et al.* 2019).

### Data Availability

Raw CCS reads have been deposited to the National Center for Biotechnology Information under BioProject number PRJNA633906. Genotypic Data from the resulting KASP assay and phenotypic data are provided in Supplementary Table S2. VCF files, Python code, and R scripts are available at the following github repository: github.com/Bassil-Lab/Zurn-et-al-2020-G3-Blackberry-SSC. KASP assays (Supplementary Table S3) are available through LGC. Supplemental material available at figshare: https://doi.org/10.25387/g3.12767864.

## Results

### Phenotypic Data

A high degree of variability was observed among progeny from the UA and USDA-ARS HCRU breeding programs (Supplementary Table S1). For the six UA populations evaluated in this study, SSC ranged from 5.3 – 14.8 °Brix in 2015 and from 4.6 – 16.2 °Brix in 2016. The mean SSC for the UA progeny was 9.9 and 9.8 °Brix in 2015 and 2016, respectively. Soluble solids content in the eight USDA-ARS HCRU populations ranged from 6.0 – 20.2 °Brix in 2015 and 5.8 – 18.9 °Brix in 2016. The mean SSC for the USDA-ARS HCRU populations was 12.4 and 11.7 °Brix in 2015 and 2016, respectively.

### Rubus SSC Candidate Gene Identification and Bait Design

Among the *Fragaria* genes in the three conserved syntenic regions (table 2), seven genes were identified with functions associated with sweetness, including beta-amylase 3 and sugar transport genes. A BLAST-search of the seven *Fragaria* genes and the 789 *Malus* sweetness-associated genes against the *Rubus* RefTrans v2 transcripts identified 279 unique genes putatively associated with sweetness in *Rubus*. Mapping these genes to the *R. occidentalis* v1.1 reference genome identified 2,122 exon sequences with start and stop boundaries. Arbor Biosciences designed 9,355 baits with 2X tiling density for 2,114 of the 2,122 exon sequences (99.6% of the exons). Despite having eight exons with no baits designed, total target region coverage was still high at 98.8% of the total length of the submitted exons.

### Sequencing and Polymorphism Identification

Sequencing and filtering of the captured genomic reads for the 40 samples produced 430,167 high-quality CCS reads (Table 1). The number of CCS reads were variable and ranged from 62 to 37,369 reads per individual. The mean and median read lengths were 2,661 and 2,610 nucleotides, respectively. The quality of the CCS reads was high and a mean phred score of 40 was observed. A total of 929,430 and 1,324,854 loci were found that had alleles different from the reference when mapping CCS reads to the ‘Hillquist’ v1 and *R. occidentalis* v3.0 assemblies, respectively.

### Identifying Polymorphisms Associated With SSC

After filtering on missing data, 12,945 and 15,194 loci were available for investigation that were identified in the ‘Hillquist’ V1 and *R. occidentalis* v3.0 genome assemblies, respectively. After the first round of statistical testing, 467 loci from the ‘Hillquist’ V1 assembly and 312 loci from the *R. occidentalis* v3.0 assembly were identified to be significant (α = 0.05).

**Figure 2:**
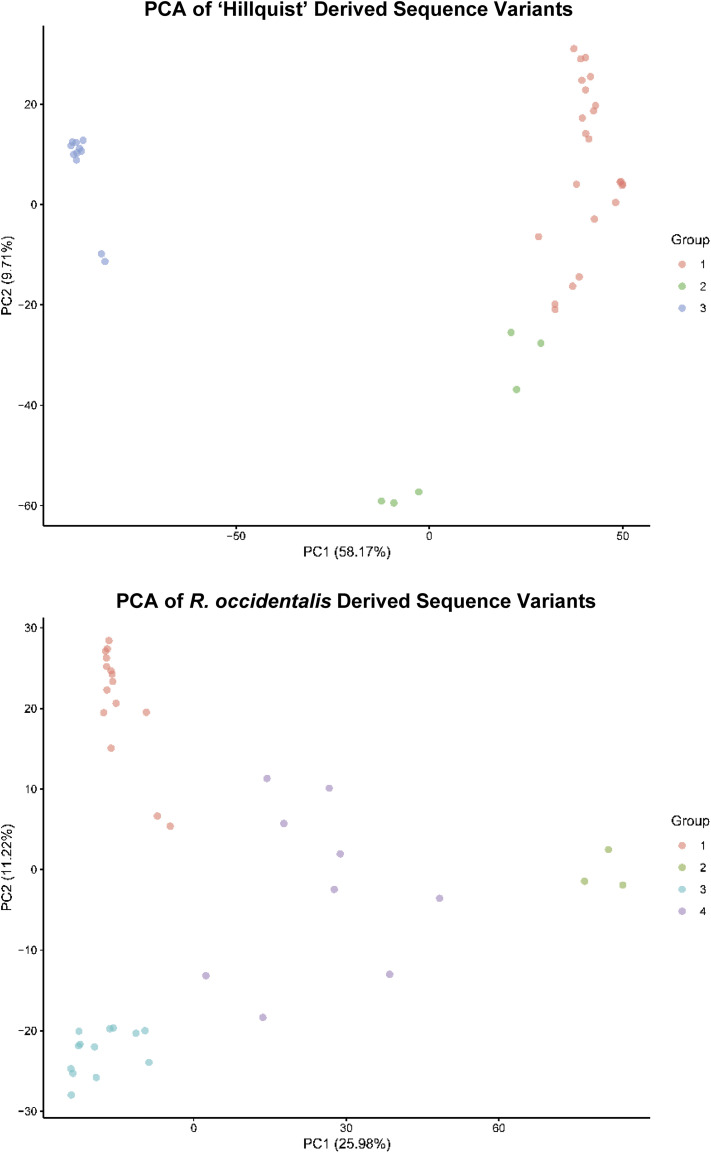
Principal Component Analysis (PCA) results of sequence variants with less than 20% missing data identified in 40 sequenced blackberry cultivars and advanced selections using the ‘Hillquist’ blackberry v1 and ORUS 4115-3 black raspberry *R. occidentalis* v3.0 assemblies. Discriminant analysis of principal components identified three and four groups for the ‘Hillquist’ and *R. occidentalis* derived variants, respectively.

The sequenced individuals were clustered into three and four sub-groups during a discriminant analysis of principal components using loci identified in the ‘Hillquist’ V1 and *R. occidentalis* v3.0 assemblies, respectively (Figure 2). Population structure was used to model the previously identified significant loci to determine independence of population structure and if allele effects differed between groups. No allele effect differences (α = 0.05) were identified between groups when using model 1, indicating that the effects of the identified loci were independent of population structure. Model 2 identified 64 alleles identified from the ‘Hillquist’ V1 assembly that were significantly (α = 0.05) associated with SSC regardless of population structure. For loci identified using the *R. occidentalis* v3.0 assembly, 109 alleles were found to be significant (α = 0.05) regardless of population structure.

### Diagnostic Marker Validation

A total of 111 KASP assays (Supplementary Table S3) representing 92 loci could be designed for the 173 significant loci identified from the two assemblies. Low GC content, dimer formation, low/high annealing temperature, or large amounts of sequence variation near the target polymorphism prevented primer design for the unrepresented targeted loci. Twenty-seven of the markers (24.3%) performed poorly or were monomorphic and were subsequently removed. Evaluation of the remaining 84 markers (Supplementary Table S2) for their association with SSC in the UA and USDA-ARS HCRU offspring populations during the 2015 and 2016 growing seasons identified a total of 48 alleles that remained significant after validation (Supplementary Table S4). Overall, most of the alleles identified had a negative influence on SSC, with only 16 being associated with an increase in SSC. Fewer alleles were found to be associated with SSC in the UA populations compared to the USDA-ARS HCRU populations.

**Figure 3:**
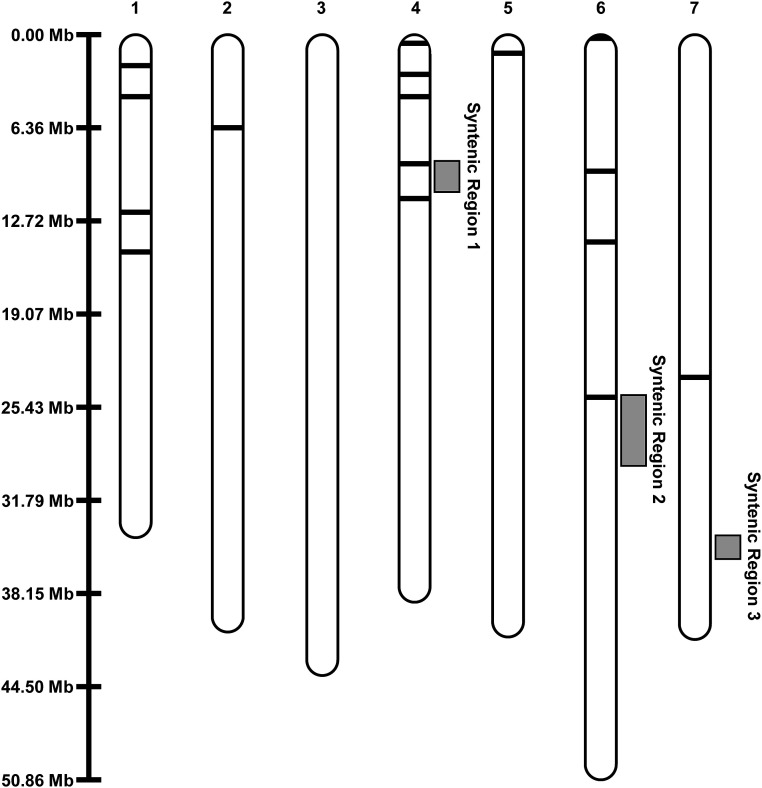
Significant SSC-associated marker positions relative to the three syntenic QTL regions (Table 2) in the black raspberry ORUS 4115-3 genome (*R. occidentalis* v3.0 assembly). The synteny viewer tool on the Genomic Database for the Rosaceae (GDR; https://www.rosaceae.org/) is suggested for an in-depth view of the synteny for these regions.

The 48 significant alleles identified in each assembly mapped to 16 regions across six of the seven chromosomes (all but Ro03) in the *R. occidentalis* v3.0 assembly (Figure 3; Supplementary Table S4). Markers associated with SSC were found in syntenic region 1 on chromosome 4 and syntenic region 2 on chromosome 6. The markers found in these regions were only significant in one environment (Oregon 2016) with the exception of marker BBS_SNP29, which was significant in two environments (Oregon 2015 and Oregon 2016; Supplementary Table S4). Three alleles in markers BBS_SNP45, BBS_INDL31, and BBS_SNP46 were found in a 736 bp region on chromosome 1 that were significant in three environments (Supplementary Table S4). The QTL associated with these markers accounted for a 1.46 °Brix difference in SSC and was named qSSC-Ruh-ch1.1. Additionally, 15 QTL regions were significant in both of the Oregon environments (Supplementary Table S4).

The genotypic data for the three significant alleles on chromosome 1 were consistent for 11 of the 13 samples that were genotyped using sequencing and KASP assays (Table 3). In two samples, ORUS 4674C and A-2487T, two KASP markers were not able to capture both diagnostic alleles that were identified during sequencing (Table 3).

**Table 3:** Genotype comparison of 13 samples that were genotyped with the KASP assay and sequencing at positions 14,978,562, 14,978,613, and 14,979,298 on Chromosome 1 from the *Rubus occidentalis* v3.0 genome assembly. Eleven of the 13 samples had the same genotypes with both methods while two samples, ORUS 4674C and A-2487T, had homozygous genotypes of the targeted allele at two and three of the positions investigated, respectively

Sample Name	Ro01 14,978,562		Ro01 14,978,613		Ro01 14,979,298	
	Sequencing	KASP	Sequencing	KASP	Sequencing	KASP
ORUS 4540A	A:T	A:T	AC:GT	AC:GT	T:A	T:A
ORUS 4647U	A:A	A:A	AC:AC	AC:AC	T:T	T:T
ORUS 4674C	A:T	T:T	AC:GT	GT:GT	T:A	T:A
Marion	A:T	A:T	AC:GT	AC:GT	T:A	T:A
Nightfall	A:A	A:A	AC:AC	AC:AC	T:T	T:T
Waldo	A:G	A:A	AC:AC	AC:AC	T:T	T:T
Bassettberry	A:G	A:A	AC:AA	AC:AC	T:T	T:T
ORUS 4647L	A:A	A:A	AC:AC	AC:AC	T:T	T:T
ORUS 4647M	A:A	A:A	AC:AC	AC:AC	T:T	T:T
ORUS 4647R	A:A	A:A	AC:AC	AC:AC	T:T	T:T
Silvan	A:A	A:A	AC:AA	AC:AC	T:T	T:T
Osage	A:T	A:T	AC:GT	AC:GT	T:A	T:A
A-2487T	A:T	T:T	AC:GT	GT:GT	T:A	A:A

### Characterization of Chromosome 1 QTL

All three significant SSC-associated alleles in qSSC-Ruh-ch1.1 were associated with a single ohnolog. The physical gene space for the three alleles in the 736 bp region on chromosome 1 contained two overlapping genes: maker-Ro01-snap-gene-149.62 and maker_Ro01_snap_gene-149.66. Maker-Ro01-snap-gene-149.62 was found to have protein domains associated with aldo-keto reductase, glycosyltransferase, or sucrose synthase genes. Maker_Ro01_snap_gene-149.66 and the haplotype sequences had domains associated with glycosyltransferase and sucrose synthase genes. A BLAST-search using both genes and the associated haplotype identified sucrose synthase genes in rosaceous species as the candidate with the highest proportion of identity (94%) and lowest e-values (0). The gene maker-Ro01-snap-gene-149.62 is possibly a chimeric sequence generated during assembly or a pseudogene because it had domain hits to two different genes, contained a small number of short exons (6), and was mostly composed of large introns. The three alleles were likely associated with maker_Ro01_snap_gene-149.66. This gene contains 14 introns and 15 exons in the *R. occidentalis* v3.0 assembly. The first two alleles were located at positions 14,978,562 and 14,978,613 in the fourth intron of maker_Ro01_snap_gene-149.66 while the third allele was located in the fifth intron at position 14,979,298.

## Discussion

The Hyb-Seq approach together with exploiting synteny among Rosaceae species effectively identified candidate genes for sweetness in blackberry. This approach captured targeted genomic sequences used to detect polymorphisms among breeding individuals and identified 173 alleles for investigation. This experiment can serve as a model approach to rapidly create tools useful for MAS in systems with scarce genomic resources, which was the intent of the RosBREED project (Iezzoni *et al.* 2017). One advantage of the approach was using PacBio sequencing in conjunction with Hyb-Seq. At low coverage, PacBio sequencing is somewhat error-prone (Jiao *et al.* 2013; Westbrook *et al.* 2015; Frank *et al.* 2016). This flaw can be overcome by sequencing the same circularized DNA molecule multiple times to form accurate consensus sequence (Jiao *et al.* 2013; Westbrook *et al.* 2015; Frank *et al.* 2016). This approach substantially reduces base call errors and significantly improves read accuracy (Jiao *et al.* 2013). Reducing sequence errors is especially important when identifying polymorphisms associated with traits because base errors can be incorrectly classified as polymorphisms. The average CCS Phred quality score in the present study was high at 40, so the likelihood that a base call error affected downstream analysis is therefore low. Moreover, the long reads generated by PacBio sequencing enabled identification of individual haplotypes within each of the sequenced samples. Considering the variable ploidy and the complex genetics found in cultivated blackberry (Clark and Finn 2011), the haplotypes present at a locus can be difficult to identify and define with short-read sequencing. To reduce consensus sequence complexity and increase primer design success, identification and preference of individual haplotypes that contained the target allele was important for diagnostic marker development.

Despite identifying 173 target alleles, KASP assays could only be developed for 92 alleles. This was mainly caused by additional polymorphisms being situated near the target allele or low GC contents. Polymorphisms near the allele of interest might explain why KASP markers BBS_SNP45, BBS_INDL31, and BBS_SNP46 designed for positions Ro01 14,978,562, Ro01 14,978,613, and Ro01 14,979,298, respectively, were only able to capture the target diagnostic allele in some of the 13 samples investigated (Table 3). Viewing the physical positions through IGV confirmed that reads from some of the 40 samples possibly representing the non-target allele contained the start of a major insertion or deletion within the flanking 25 base pairs of the target. These polymorphisms could interfere with annealing of the forward KASP primer of the non-target alleles, producing only homozygous calls for the target alleles in a sample. It is also possible that off-target amplification could have influenced the scoring of these markers, given how large and conserved some of the targeted gene families are. Markers may have amplified paralogous sequences in addition to the target locations. Given the complexity of the blackberry genome, it would be difficult to separate these off-target amplification events from on-target amplification of ohnologous loci. Therefore, the KASP assays in these cases can only be used to assess the presence or absence of the target allele and should not be used to assign dosage. For the 81 alleles associated with SSC for which a KASP marker assay could not be designed, sequencing-based genotyping methodologies may be the only effective way to validate these alleles.

The 48 significant markers identified in this study were located in 16 regions in the *R. occidentalis* v3.0 genome assembly (Figure 3); many of these regions were only significant in a single testing environment (Supplementary Table S4). This outcome is not unexpected and has been observed during SSC studies in other rosaceous crops (Etienne *et al.* 2002; Zorrilla-Fontanesi *et al.* 2011; Lerceteau-Köhler *et al.* 2012; Verma *et al.* 2017). Fruit SSC is known to have low heritability and individual SSC QTL often explain less than 10% of the phenotypic variation (Etienne *et al.* 2002; Zorrilla-Fontanesi *et al.* 2011; Lerceteau-Köhler *et al.* 2012; Verma *et al.* 2017). More QTL were identified in the USDA-ARS HCRU than in the UA breeding program. The USDA-ARS HCRU breeding program releases cultivars of varying ploidies for the processing market and features trailing germplasm from Western North America and erect and semi-erect germplasm from Eastern North America. In contrast, the UA breeding program has historically focused on erect germplasm from Eastern North America for the fresh market (Clark and Finn 2011). The differences in market focuses has caused the USDA-ARS HCRU breeding program to focus on higher acid and sugar content while the UA program has placed emphasis on postharvest storage and transportation capacity (Clark and Finn 2011). As such, the increased number of QTL may be associated with the increased diversity of the germplasm found in the USDA-ARS HCRU breeding program. The markers BBS_SNP45, BBS_INDL31, and BBS_SNP46 identified on chromosome 1 compose a QTL that was detected in three environments and accounted for a 1.5 °Brix increase in SSC. This QTL, qSSC-Ruh-ch1.1, was detected in germplasm from both the UA and the HCRU breeding programs. qSSC-Ruh-ch1.1 is expected to be quite stable as these two breeding programs were reported to have very genetically distinct germplasm driven by geographical differences (Zurn *et al.* 2018).

The gene space for qSSC-Ruh-ch1.1 was identified, and all three alleles were located in introns flanking the fifth exon in the gene marker-Ro01-snap-gene-149.66 from the *R. occidentalis* v3.0 assembly. Conserved domain and BLAST searches for the genes associated with the QTL revealed highest homology to sucrose synthase (SUS) genes from the glycosyltransferase-4 subfamily of the glycosyltransferase super family (reviewed by Stein and Granot 2019). In plants, many genes including SUS genes have been reported to control the accumulation of sugars and starch in plants such as Arabidopsis, rice, maize, and apple (Tsai *et al.* 1970; Perez *et al.* 1975; Reidel *et al.* 2008; Rennie *et al.* 2012; Raynaud *et al.* 2016; Wang *et al.* 2018). Sucrose synthase genes catalyze the reversible cleavage of sucrose into fructose and either uridine diphosphate glucose (UDP-G) or adenosine diphosphate glucose (ADP-G). Plant SUS genes are divided into three separate clades (SUS I, SUS II, and SUS III), are ubiquitous in monocots and dicots, and range widely in number among species. In rosaceous crops, the number of SUS genes vary from six in peach (*Prunus persica*; Zhang *et al.* 2015) to 11 in apple (*Malus ×domestica*; Tong *et al.* 2018) and black raspberry (*R. occidentalis*; Van Buren *et al.* 2018) and 30 in Chinese pear (*Pyrus* ×*bretschneideri*; Abdullah *et al.* 2018). Isoforms of SUS genes in the Rosaceae are differentially expressed in different tissues (Zhang *et al.* 2015; Zhao *et al.* 2017; Abdullah *et al.* 2018). Some isoforms are expressed during fruit development in pear (*PbSS5*, *PbSS3*, and *PbSS24*; Abdullah *et al.* 2018) and strawberry (*FaSS*1; Zhao *et al.* 2017). In strawberry, downregulation of *FaSS*1 significantly delayed fruit ripening and resulted in decreased sucrose content (Zhao *et al.* 2017), indicating a possible role in controlling fruit sweetness. Sucrose synthase, sucrose-phosphate synthase (SPS), and acid invertase activity have also been analyzed for their effects on SSC and sugars in Asian pear fruit (Moriguchi *et al.* 1992). Sucrose synthase transcript levels and sucrose content were highly correlated in 23 Asian pear cultivars. The true function of this haplotype in blackberry is unknown due to scarce genomic resources in *Rubus* and a lack of functional understanding of glycosyltransferase genes and their role within different plant species (Tong *et al.* 2018; Thirugnanasambandam *et al.* 2019). Still, synteny and gene conservation within Rosaceae suggest that this *Rubus* SUS gene imparts similar sugar metabolic function as pear and strawberry SUS genes.

When examining where the syntenic regions mapped to on the *R. occidentalis* v3.0 assembly, very few significant markers were identified that were associated with these regions (Table 2; Figure 2). Thirteen markers were associated with syntenic region 1 (Figure 2; Supplementary Table S4). Diagnostic alleles for three of these markers (BBS_SNP23, BBS_SNP24, and BBS_SNP88) were associated with an increase in SSC while the remaining 10 were associated with a decrease in SSC. The marker BBS_INDL12 on chromosome 6 also appeared to be associated with syntenic region 2, with the presence of the diagnostic allele being associated with a 1.4 °Brix increase in SSC. In each of these cases, the significant effect was only detected in a single environment. The presence of associated markers in the syntenic regions suggests that QTL for conserved pathways may be transferable across genera within a family. Given these markers were significant in only a single environment, additional testing and possibly gene cloning is needed to confirm the transferability across genera of the associated QTL. Traits associated with flower petal formation and fruit aromatics and flavor components could be investigated in the future. While tempting, disease resistance QTL, with the exception of MLO-mediated powdery mildew resistance (reviewed in Kusch and Panstruga 2017), should be avoided for this approach as unique host-pathogen interactions are expected to govern the evolution of resistance genes within individual species.

Genomic tools are rapidly being developed for many agriculturally important crops. Despite this, a number of regionally important crops remain that lacks the resources available to agronomic crops. The genomics resource deficiency of cultivated blackberry is fortunately beginning to be overcome with exploitation of large genomics resources available for related crops such as apple, peach, and strawberry. Such resources can be leveraged to target genes associated with pathways conserved at a family level. Using family-level information, genomic and bioinformatics scientists can develop new tools to assist breeders for crops that have not yet benefited from the genomics revolution.
